# A molecular spectroscopy approach for the investigation of early phase ochronotic pigment development in Alkaptonuria

**DOI:** 10.1038/s41598-021-01670-z

**Published:** 2021-11-19

**Authors:** Andrea Bernini, Elena Petricci, Andrea Atrei, Maria Camilla Baratto, Fabrizio Manetti, Annalisa Santucci

**Affiliations:** Department of Biotechnology, Chemistry and Pharmacy, University of Siena, via Aldo Moro 2, Siena, Italy

**Keywords:** Biochemistry, Chemical modification

## Abstract

Alkaptonuria (AKU), a rare genetic disorder, is characterized by the accumulation of homogentisic acid (HGA) in organs due to a deficiency in functional levels of the enzyme homogentisate 1,2-dioxygenase (HGD), required for the breakdown of HGA, because of mutations in the HGD gene. Over time, HGA accumulation causes the formation of the ochronotic pigment, a dark deposit that leads to tissue degeneration and organ malfunction. Such behaviour can be observed also in vitro for HGA solutions or HGA-containing biofluids (e.g. urine from AKU patients) upon alkalinisation, although a comparison at the molecular level between the laboratory and the physiological conditions is lacking. Indeed, independently from the conditions, such process is usually explained with the formation of 1,4-benzoquinone acetic acid (BQA) as the product of HGA chemical oxidation, mostly based on structural similarity between HGA and hydroquinone that is known to be oxidized to the corresponding para-benzoquinone. To test such correlation, a comprehensive, comparative investigation on HGA and BQA chemical behaviours was carried out by a combined approach of spectroscopic techniques (UV spectrometry, Nuclear Magnetic Resonance, Electron Paramagnetic Resonance, Dynamic Light Scattering) under acid/base titration both in solution and in biofluids. New insights on the process leading from HGA to ochronotic pigment have been obtained, spotting out the central role of radical species as intermediates not reported so far. Such evidence opens the way for molecular investigation of HGA fate in cells and tissue aiming to find new targets for Alkaptonuria therapy.

## Introduction

Homogentisic acid (2,5-dihydroxyphenylacetic acid, HGA) is a para-diphenol carboxylic acid widely diffused in animals, plants, and unicellular organisms. It was discovered in the nineteenth century but depth of knowledge on this compound grew when the substance isolated from urine by Boedeker in 1859^1^ was subsequently shown by Wolkow and Baumann^2^ to be HGA. A strong link was next built between HGA and the ochronotic pigment found in the urine of alkaptonuric patients^3^.

HGA belongs to the metabolic pathway of phenylalanine and tyrosine. It is a precursor of plastoquinone and tocopherols in plants and is involved in the machinery designated to protect chlorophyll from sunlight degradation. In microorganisms, such as bacteria^4–7^, fungi^8,9^, and yeasts^10^, as well as in mammals^11^, HGA is responsible for pigmentation due to the formation of pyomelanin-based polymers or oxidative dimerization in normal or stress conditions.

In human metabolism, HGA is found in the subpathway that synthesizes acetoacetate and fumarate from L-phenylalanine, in particular in the step catalysed by homogentisate 1,2-dioxygenase (HGD) transforming the acid into 4-maleylacetoacetate. Accumulation of HGA, leading to ochronotic pigment and development of Alkaptonuria^12,13^, is related to HGD gene mutations^14^ disrupting the enzyme or altering its catalytic activity in multiple ways^15–17^.

From a chemical point of view, HGA is the reduced form (termed quinol or hydroquinone) of 1,4-benzoquinone acetic acid (BQA). Oxidation of HGA is dependent on solvent, temperature, and pH. It occurs faster in alkaline solutions, while it is retarded in acidic conditions. Electrochemical studies demonstrated that a two-proton-two-electron reaction^18^ is responsible for the transformation of HGA (0.8 mM in 0.1 M phosphate buffer) into the corresponding oxidized form, with a redox potential of 0.636 V versus the standard hydrogen electrode, in an aqueous solution at 25 °C^19^. Many previous studies showed that UV absorption spectra of synthetic HGA and BQA are characterized by a peak at 290 and 250 nm, respectively^20^. Exposure to air (that is, reaction with oxygen) led to a non-enzymatic time-dependent disappearance of the peak at 290 nm and appearance of that at 250 nm (and an additional peak above 315 nm). Additional exposure to oxygen or treatment with alkali solutions (strong bases such as NaOH, and weak bases such as NH_3_) led to darkening the aqueous media where the formation of complex structures derived from HGA polymerization have been hypothesized by different research groups. Recently, additional absorption peaks at 406 and 430 nm were recorded in both alkaptonuric urine and HGA solution about 1 min after treatment with alkali^21^. Treatment with strong alkali resulted in time-dependent absorbance values higher in comparison to those found with ammonia, while the addition of sodium hypochlorite accelerated HGA oxidation^22^.

One of the most accredited past hypotheses about the fate of HGA in pathological conditions (such as alkaptonuria) was oxidation to BQA and subsequent polymerization to form a pyomelanin-like polymer that accumulates in joints and cartilage. Experimental evidence was also provided to demonstrate the ability of BQA to react with biological amines^23^ and, thus, to justify the collagen denaturation found in alkaptonuric patients. Moreover, the formation of an ochronotic-like pigment in connective tissue was also hypothesized as a consequence of a copper (II)-catalyzed oxidation of HGA mediated by an HGA polyphenol oxidase^24^. Involvement of metal ions in the redox equilibrium between HGA and BQA suggested the possible role of radicals within the same reaction. It was reported that autoxidation of HGA to BQA also led to the formation of reactive oxygen species, such as hydrogen peroxide and superoxide^25^, that in turn contributed to the formation of the ochronotic pigment and impairment of connective tissue in alkaptonuric patients^26^.

The structural properties of the HGA-derived pyomelanin were investigated by many research groups. The molecular weight of about 10–14 kDa suggested dozens of HGA monomers in the pyomelanin structure, while infrared analysis provided evidence only for expected chemical groups and fragments^27^. Examples of the polymers derived from oxidative coupling of HGA in alkaline solutions are represented by linear polyhydroquinone structures with their phenyl rings directly linked to each other or through a bridged oxygen atom^28^.

Although many experimental reports showed that BQA represented the product of HGA chemical oxidation, no scientifically compelling evidence was provided to support this hypothesis. The formation of BQA from HGA by oxidation reaction was instead formulated based on structural similarity between HGA and hydroquinone that is known to be oxidized to the corresponding para-benzoquinone.

Electrochemical experiments in glassy carbon electrodes showed that aqueous solutions of HGA are stable when pH < 8^19^. At higher pH values, HGA underwent oxidation that was reversible in electrochemical experiments. On the contrary, when pH > 11, chemical oxidation of HGA by atmospheric oxygen is as fast as that the UV spectrum of HGA lacked the expected peak at 290 nM and the peak corresponding to the oxidized form of HGA (248 nM) decreased during the time, thus suggesting further degradation. A very intriguing hypothesis on the structure of the compounds generated from the reaction of the oxidized HGA was based on the oxidative ring opening of quinones that can lead to oxalic acid. Further electrochemical experiments demonstrated that oxidation of HGA in alkaline solutions involved atmospheric oxygen and singlet oxygen species. Moreover, based on the knowledge that oxidative ring-opening of para-quinones yielded (substituted) maleic acid, one of the compounds resulting from HGA oxidation can be represented by 1-propene-1,2,3-tricarboxylic acid (aconitic acid).

Recently, results on pigments generated on fresh or stored aqueous solutions of HGA alone or in the presence of NaOH and tyrosine were reported^29^. HGA alone or added with dilute NaOH solutions (0.1 M) did not show the formation of polymeric pigments (although the solutions became coloured) and maintained the original HGA concentration. Increasing the NaOH concentration to 1.0 M, HGA levels decreased to disappearance within 10 days, yielding a pigment with a high molecular weight that showed the same FT-IR properties of the pigment found in stocked solutions. In particular, peaks of HGA solutions were not conserved, except for that of the carboxy carbonyl group (1690 cm^−1^). Free phenol OH signals (3480 cm^−1^) are not present and are replaced by peaks of the corresponding hydrogen-bonded groups (3300 cm^−1^). Unexpectedly, it was suggested that the absence of peaks of aromatic C-H bonds (900–700 cm^−1^) could be accounted for by C–C and C–O bonds between aromatic units. FT-IR spectra also showed that the presence of tyrosine did not affect the transformation of HGA into the pigment. Finally, no proton and carbon signals were obtained by NMR techniques on the pigmented polymer.

In summary, there is a convergent opinion among recent and past literature that HGA undergoes oxidation to BQA that in turn evolves to a polymeric pigment of undefined structure. On the contrary, divergent conclusions have been reported about the stability of the sole HGA in an aqueous solution and human urine.

In the attempt to shed further light on the mechanisms regulating HGA transformation into the polymeric pigment found in alkaptonuric patients, a series of experimental techniques has been applied to aqueous solutions of commercially available HGA at different concentrations in different conditions.

## Materials and methods

### Biological samples

Adult patients (five females and five males ranging 42–69 years) were studied after the clinical diagnosis of AKU was established. None of the patients was under specific treatment. Age-matched controls of both sexes with no arthropathies or metabolic disorders were also analysed (n = 20). Urine samples were obtained from patients and controls, frozen just after collection and stored at -80 °C. Patients and controls gave written informed consent before inclusion collection. The whole study was conducted following the approval of the Siena University Hospital Ethics Committee. The informed consent and protocols conformed to the standards set by the latest revision of the Declaration of Helsinki.

### NMR spectroscopy

Urine samples were prepared by adding a stock phosphate buffer (Merck KGaA) to the biofluid to a final concentration of 150 mM to obtain a pH of 7.4; 0.5 mM DSS (sodium 2,2-dimethyl- 2-silapentane-5-sulfonate-d6, Cambridge Isotope Laboratories, Canada) was used as the internal standard for both chemical shift referencing and peak intensity calibration; the sample final volume was 700 μL with 10% D2O (Cambridge Isotope Laboratories, Canada). Pure HGA samples were prepared in the same conditions as the biofluid, with a final concentration of 5 mM and 30 mM for the molecule. All NMR spectra were acquired on Bruker Avance 600 MHz. ^1^H spectra were acquired over a spectral width of 12,000 Hz and digitalized over 32K points, 32 transients were recorded with a repetition delay of 4 s using a standard Bruker NOESY ^1^D sequence with presaturation (NOESYPR1D). ^13^C spectra were acquired over a spectral width of 36,000 Hz and digitalized over 64K points, 1024 transients were recorded with a repetition delay of 5 s using a standard Bruker 1D sequence with power-gated decoupling sequence or J-modulated spin-echo to determine the number of attached protons. Alkalinisation of the samples was achieved by adding a concentrate stock solution of NaOH to the NMR tube to a final concentration of 125 mM. Each sample was replicated twice as well as the alkalinisation experiment.

### EPR spectroscopy

CW (Continuous wave) X-band (9 GHz) EPR spectra of 45 μL HGA 30 mM and 5 μL NaOH 1 N were recorded at room temperature. The reaction was monitored at different reaction times after the addition of reagents. The reaction was also tested at acidic pH after the addition of HCl 1 N and in buffer solution at pH = 8. EPR measurements were performed with a Bruker E580 Elexsys Series using the Bruker ER4122SHQE cavity filling in a 1 mm ID quartz capillary tube and then it was placed inside standard suprasil EPR tubes. EPR spectra simulations were performed with the Easyspin software package^30^, using the "garlic function".

### DLS measurements

A Zetasizer NanoZS90 instrument (Malvern, Worcestershire, UK) was used for the DLS measurements. The scattering angle was 173° and the measurements were performed at 25 °C. The stock solution of the reacted HGA was diluted with double distilled water.

### BQA preparation

*Synthesis of 2-(3,6-dioxocyclohexa-1,4-dien-1-yl)acetic acid* (**3**): 4-Iodophenoxy acetic acid **2** (7 mg, 0.025 mmol) was dissolved in a 2:1 mixture of CF_3_CH_2_OH/H_2_O (5 mL) under N_2_. Oxone® (1.23 g, 4 mmol) and 2,5-dimethoxyphenylacetic acid **1** (100 mg, 0.5 mmol) were added and the reaction mixture and stirred in the dark at r.t. under N_2_ for 4 h. The mixture was filtered and the solution extracted with CH_2_Cl_2_ (2 × 10 mL) and washed with H_2_O (2 × 5 mL). The organic phase was dried over dry Na_2_SO_4_ and the solvent evaporated under reduced pressure after filtration. BQA **3** was obtained as an orange solid in quantitative yields. ^1^H-NMR (400 MHz, CD_3_OD, δ ppm): 6.79–6.65 (m, 3H), 3.39 (s, 2H) (see Supplementary Information Fig. S4).

## Results and discussion

### NMR spectra of urine samples

**Figure 1:**
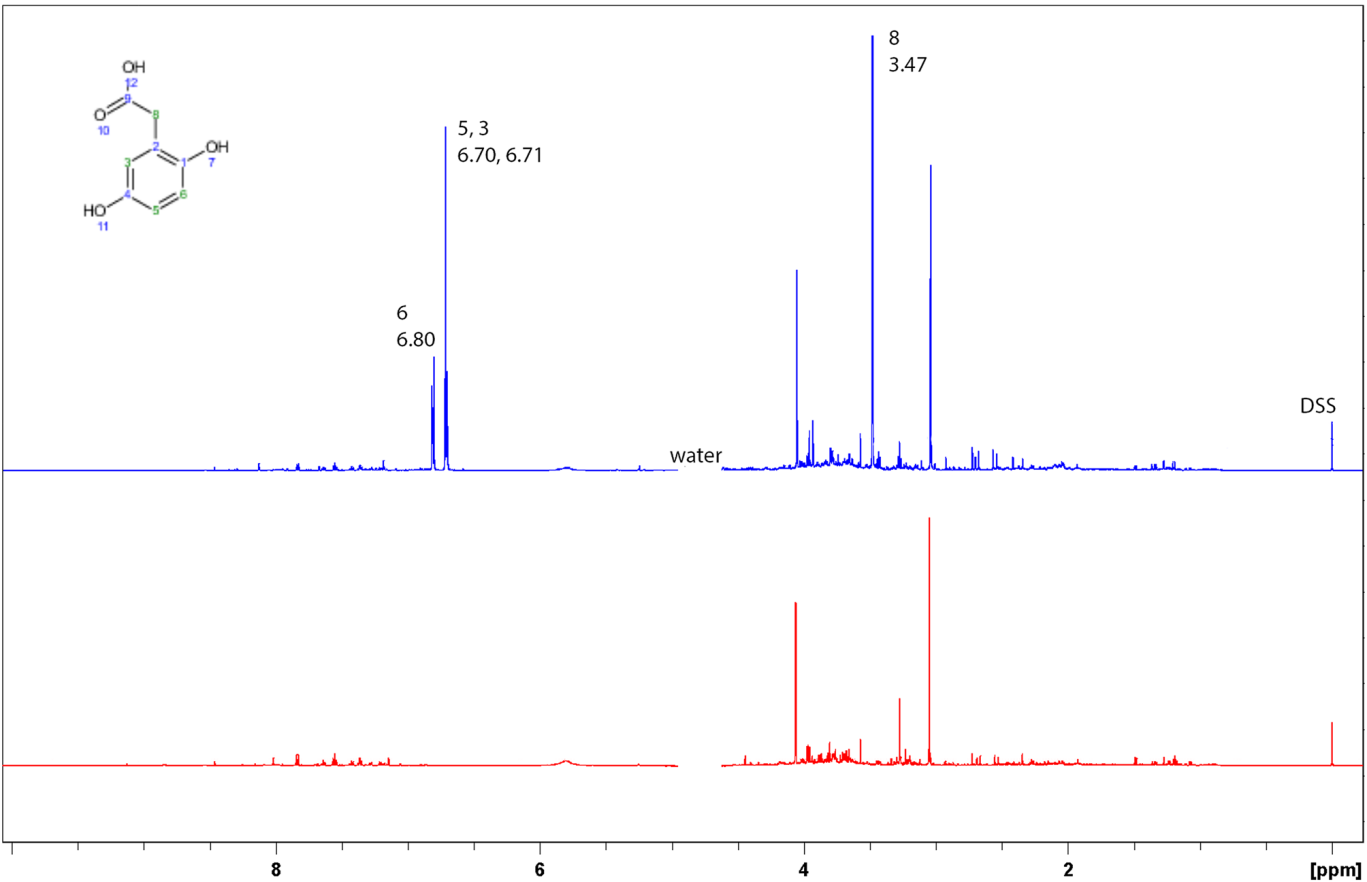
Comparison of ^1^H NMR spectra of a urine sample (top) from AKU patient (U425, HGA concentration 33.0 mM) with a urine sample (bottom) from a control (C391, no HGA).

**Figure 2:**
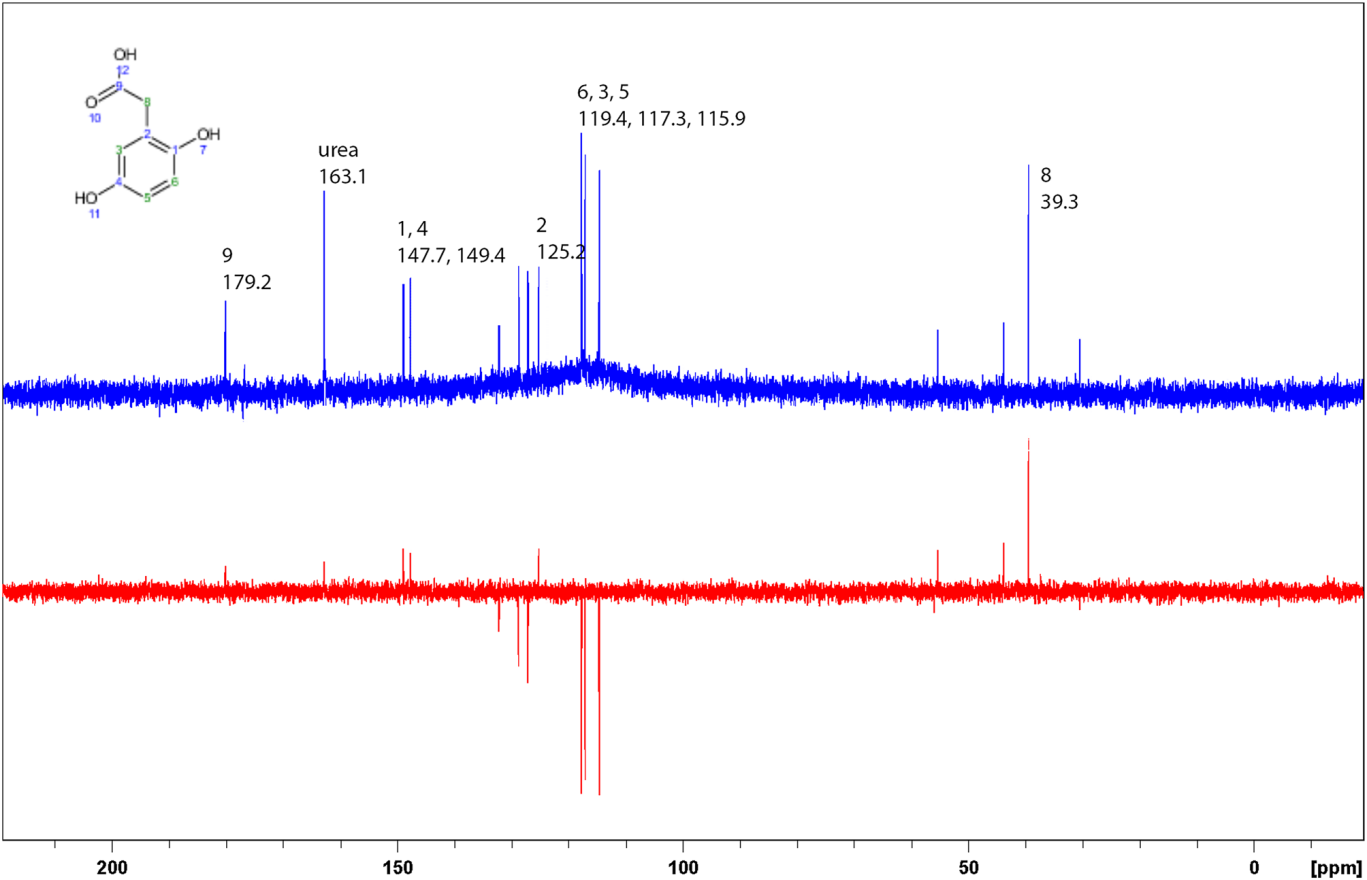
^13^C spectrum (top) of the same AKU patient urine sample shown as ^1^H spectrum in Fig. 1; the bottom spectrum is APT with positive and negative peaks for carbon bearing an even or odd number of protons, respectively, further confirming the assignment.

Urine samples of 10 AKU Italian patients (adults, 5 males, 5 females) were investigated by ^1^H and ^13^C spectroscopy. HGA peaks were unambiguously assigned through comparison with spectral resonances from HMDB^31–34^ entry HMDB0000130 in the standardized buffer and temperature conditions recommended^35^ and further confirmed by cross peak correlations in two-dimensional spectroscopy (TOCSY and ^13^C-HSQC). Spectra of 20 control urine samples were acquired under the same conditions and showed no resonances in the spectral ranges of HGA. Full assignment for both nuclei is reported in Figs. 1 and 2. Calibration with 0.5 mM internal standard DSS was used for the determination of the absolute concentration of HGA in the samples analyzed throughout the study (see results in Table 1).

**Table 1:** Urine samples analyzed in the study with relative HGA concentration as determined by ^1^H NMR (M = male, F = female).

Urine sample	HGA (mM)
364M	13.7 ± 2.4
381M	12.1 ± 1.1
385M	11.3 ± 0.8
414M	42.6 ± 0.6
425M	33.0 ± 2.3
369F	25.2 ± 2.9
370F	10.5 ± 0.9
371F	34.0 ± 3.9
413F	18.5 ± 3.3
420F	12.1 ± 5.7

### Transformation of urine upon alkalinisation

Alkalinisation of urine from AKU suspects has been used as a diagnostic method for a long time and is still reliable as a first, on-the-field test. The addition of sodium hydroxide causes urine to darken suddenly due to the rise of species conducive to the formation of ochronotic pigment. The process has been followed throughout its time evolution by NMR for the first time, starting from the alkali addition until the end of reaction as observable within the technique limits. In particular, the HGA transformation in alkalinised AKU urine samples can be accurately followed thanks to its very high concentration (tens of millimolar, see Table 1) in respect to other metabolites, resulting in peak intensities that dominate the ^1^H spectrum (see Fig. 1) allowing them to be easily followed along with the transformation. To assess the reproducibility of the in vitro chemical-induced changes of HGA upon alkalinisation, all the previously characterised AKU urine samples were added the same amount of NaOH), and time evolution of the spectra monitored at times 1’, 30’, 50’, 12 h, 16 h, 2d, 7d, 14d. Moreover, the experiment was replicated twice for each patient. All the samples showed a reproducible behaviour upon alkalinisation, described as follows.

**Figure 3:**
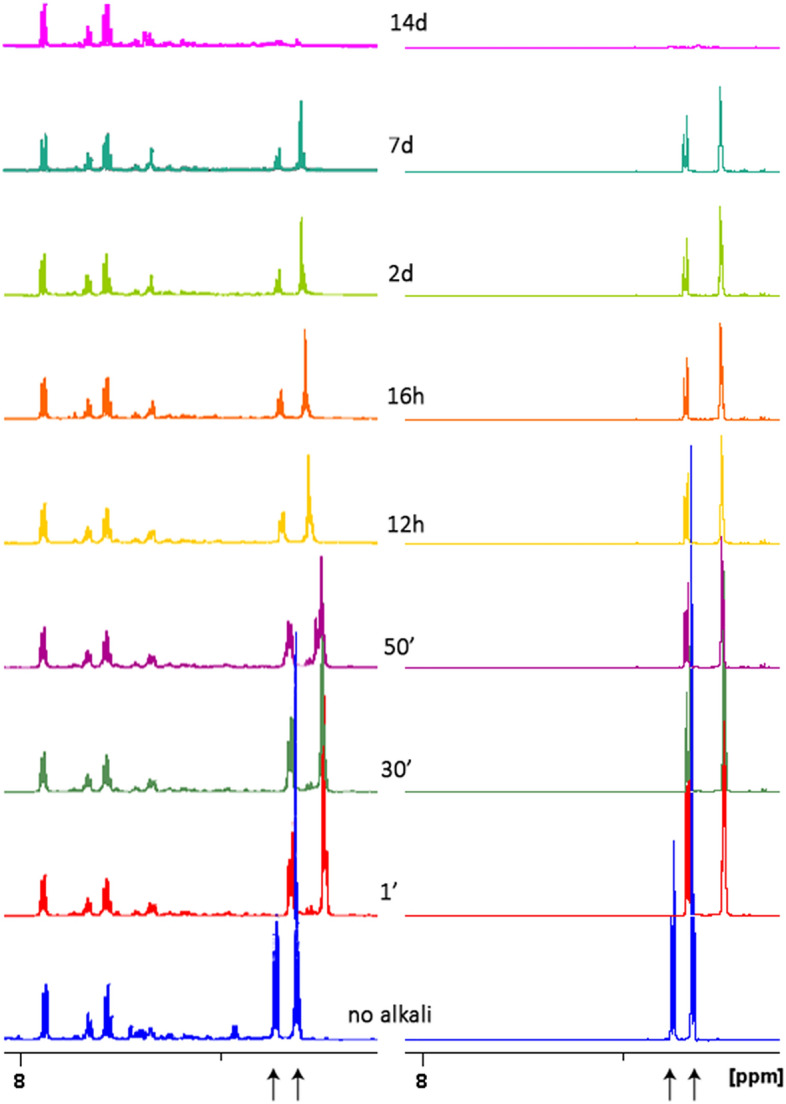
Reaction under alkaline condition monitored by ^1^H spectroscopy for a urine sample (left panel) and HGA solution (right panel) up to 14 days: from bottom to top, spectra acquired before alkalinisation, then after 1’, 30’, 50’, 12 h, 16 h, 2d, 7d, 14d, are shown (aromatic segment); the same rate of signal weakening for HGA peaks (marked with arrows) down to the noise level is apparent for both samples. HGA solution was made at the same concentration of the metabolite in the urine sample, differences in peaks shape arise from the different viscosity of the two.

NMR spectra showed a sudden upfield chemical shift change of HGA resonances upon alkali addition (see Fig. 3); consistent shift of all HGA resonances and conservation of the overall integral of the peak groups could be ascribed to the deprotonation of carboxy and hydroxy groups of the molecule, as it will be also showed by NMR titration, discussed later. Change in appearance of aromatic peak groups could be ascribed to a subtle change in the overlapping of the fine structure of the multiplets. Time evolution monitoring of the spectrum showed a slow downfield shift of the same peaks together with an exponential decrease in the integral, without the rise of other species within the observability limit of the crowded urine spectrum; on the opposite the progressive darkening of the sample is apparent. The change in the spectrum is proportional to a marked decrease in the pH, possibly explaining the downfield shift of the HGA peaks toward the original frequencies. After 14 days the HGA peaks disappeared almost completely, without giving rise to other observable signals and with conservation of the overall integral excluding the HGA resonance, and the pH stabilizes at 7.9. Such behaviour could be possibly explained with the formation of high molecular weight, slow-tumbling species or paramagnetic species, both undergoing severe signal broadening up to disappearance into the baseline; both hypotheses were investigated by mean of NMR-complementary techniques and discussed later.

**Figure 4:**
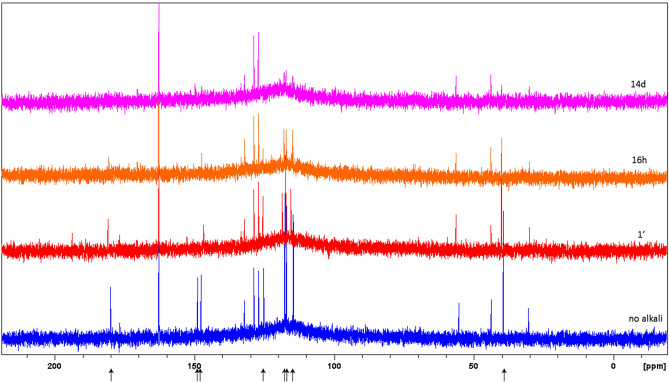
^13^C spectroscopy of a urine sample: from bottom to top, spectra acquired before alkalinisation, then after 1’, 16 h and 14d, are shown. Loss of signal intensity for HGA peaks (marked with arrows) down to the noise level is apparent.

To exclude the formation of unsaturated molecules not giving signals in ^1^H NMR, proton spectra were alternated with ^13^C spectra along the monitoring time. Carbon spectra showed the same time evolution behaviour as for proton spectra, with peaks from HGA decreasing intensity down to the noise limit and no rise of new peaks; conservation of the integral for the rest of the spectrum is also apparent (Fig. 4).

The urine spectrum is very crowded by metabolites (over 150 has been identified by ^1^H NMR^36,37^ so far) signals and, although HGA peaks are easy to follow, the formation of minor species along the HGA transformation could be hindered in the spectrum. The urine experiments were then reproduced using solutions of HGA of the same concentration under the same experimental conditions, allowing a more resolved spectrum to be obtained; interference in the reaction by other metabolites is also avoided.

Alkalinisation of a 35 mM, aqueous sample of HGA gave the same change in chemical shift as in the corresponding urine sample, and the back-shifting ratio, integral and pH decrease were the same as well, see Fig. 3. Unlike in the urine spectrum, the flat baseline (thanks to the absence of other metabolites) and the sharper peaks (thanks to the reduced viscosity), allow for better monitoring of the reaction while conserving the reaction pathway to the dark-coloured species.

### UV characterization of HGA and BQA

**Scheme 1:**
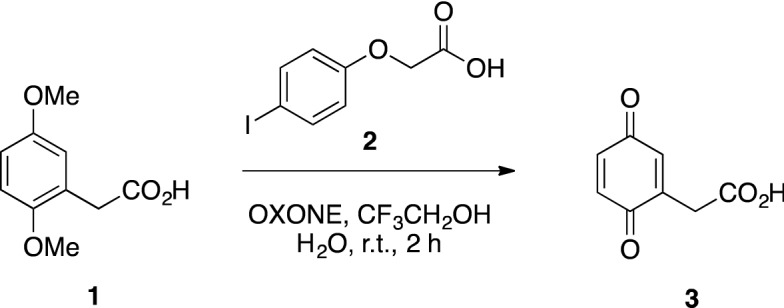
Synthesis of BQA.

**Figure 5:**
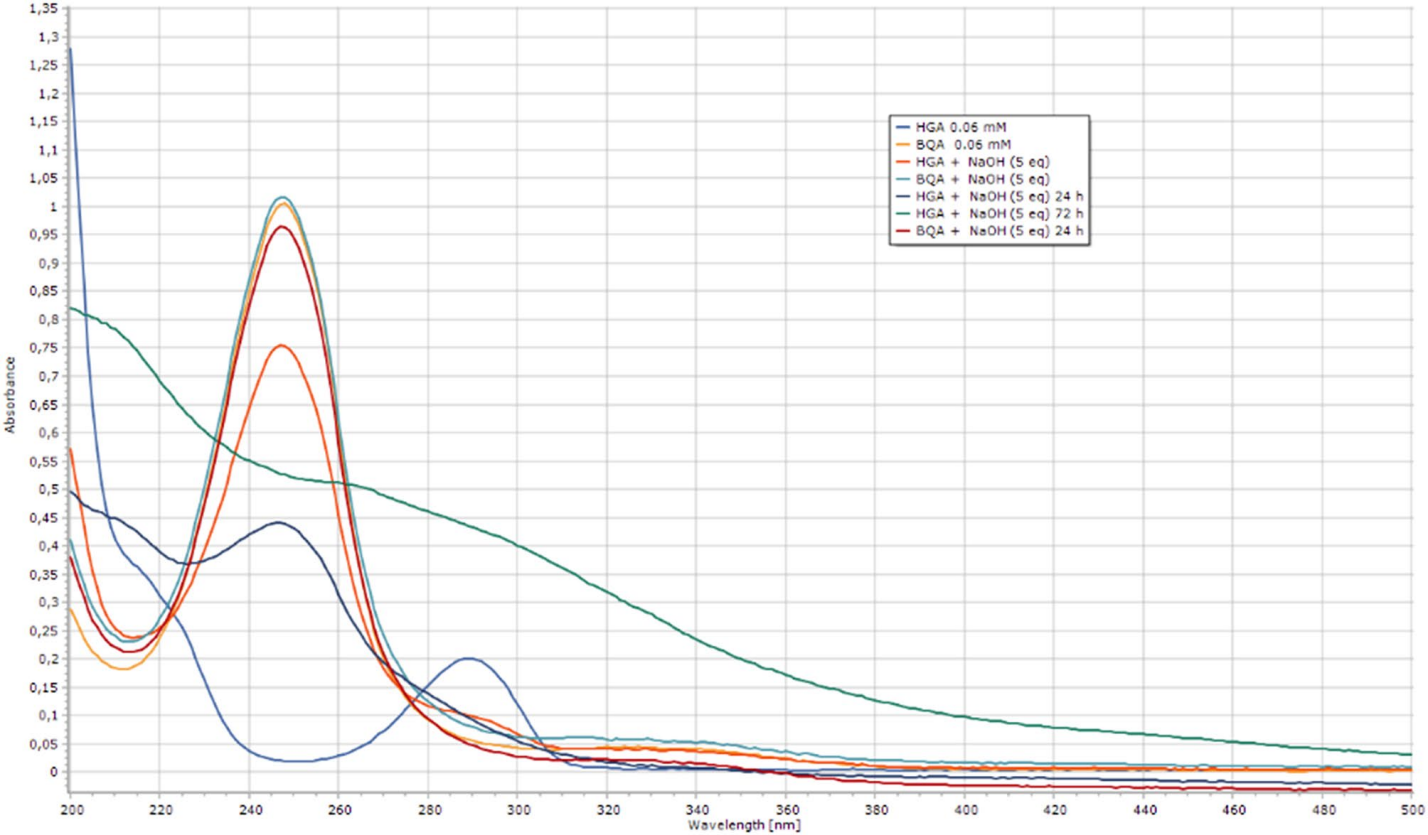
UV spectra of BQA and HGA (0.06 mM) in H_2_O and alkali.

All the data reported in the literature assumes the BQA formation by treatment of HGA with alkali. To avoid any possible mistake, the synthesis of BQA was first of all explored. 2-(2,5-Dimethoxyphenyl)acetic acid (**1**) was oxidized by treatment with hypervalent iodine in situ generated by a catalytic amount of **2** and OXONE in a mixture of trifluoroethanol and water (Scheme 1)^38,39^. BQA (**3**) was obtained in quantitative yield and characterized by ^1^H-NMR and MS analysis as a crude mixture. The product was demonstrated to be light and temperature-sensitive and need to be stored at − 80 °C. UV spectra of HGA and BQA 0.06 mM solutions in H2O were firstly recorded and compared with the corresponding UV spectra in the presence of 1 up to 5 equivalents of NaOH (Fig. 5). The data obtained are apparently in agreement with the literature suggesting BQA formation by treatment of HGA with 5 equivalents of NaOH^15^.

### NMR characterization of HGA and BQA

Nevertheless, a new scenario was disclosed once the same samples of BQA and HGA with NaOH have been analyzed by ^1^H-NMR. Particularly, HGA treatment with a 5 equivalents excess of NaOH didn’t show BQA arising.

**Figure 6:**
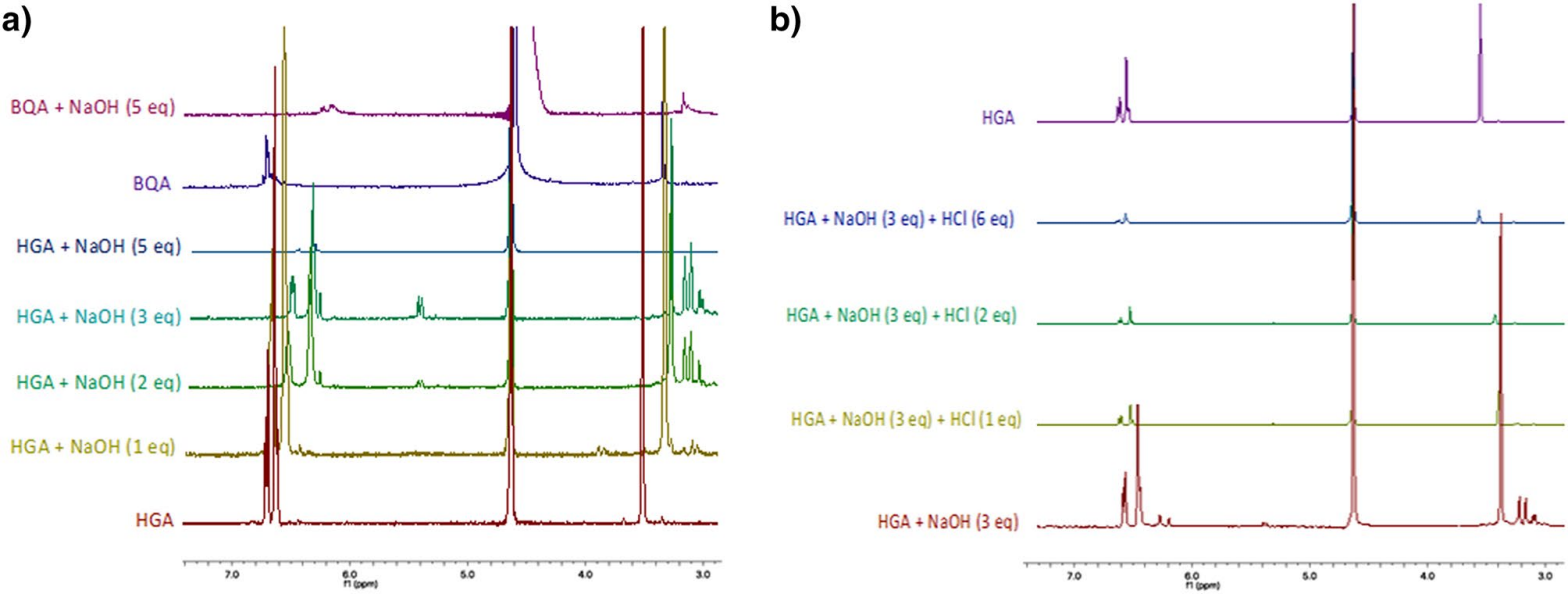
(**a**) ^1^H-NMR BQA and HGA in D_2_O/H_2_O in the presence of different equivalents (1 to 5); (**b**) titration of HGA in NaOH solution by addition of different equivalents of HCl (1 to 6).

The first reaction between NaOH and HGA was an acid–base titration (Fig. 6a) that can be reverted to HGA by treatment with HCl (Fig. 6b). The first equivalent of NaOH added to the HGA solution in D_2_O (4 mg/mL) was responsible for the carboxylic acid deprotonation followed by the phenate formation once 2 additional equivalents of alkali were added. It is interesting to note that starting from the HGA solution containing 3 equivalents of NaOH, it is possible to revert the reaction by adding 1 to 6 equivalents of HCl, thus obtaining full protonated HGA (Fig. 6b).

The product obtained by adding 5 equivalents of NaOH is different from the one formed by BQA (**3**) in the same conditions thus demonstrating that the oxidation of HGA eventually occurs in a second step (Fig. 6a).

The addition of NaOH to both HGA and BQA solutions resulted in an immediate change in the colour of the mixture from pale yellow to brown. The chemical shifts of all the signals in the ^1^H-NMR spectra change. Particularly, in HGA spectra the signals at 6.70, 6.60, 3.47 ppm shifted to 6.41, 6.28, 3.25 ppm respectively when 5 equivalents were added (Fig. 6a). By treating BQA with NaOH the multiplet at 6.74–6.65 ppm moved to 6.24–6.18 ppm while the singlet at 3.37 ppm moved to 3.20 ppm. These data were coherent with the formation of HGA and BQA salts in an alkaline medium.

On the contrary, when alkali solutions of HGA is conserved for more than 12 h, the water-soluble brown pigment that forms does not disappear upon the addition of HCl. pH values of the alkaline solution of HGA decreased by about one unit over time because of CO_2_ formation, as confirmed by the formation of a white precipitate (BaCO_3_) when Ba(OH)_2_ is used in place of NaOH. The CO_2_ elimination process suggests a free radical intermediate; to test such a hypothesis, two experiments were performed. NaOH was added to the HGA solution in the presence of the water-soluble radical scavenger Tempol^40–43^. A water solution of Tempol with HGA or NaOH didn’t show any signal in the ^1^H spectrum due to the long-range effect of the electronic relaxation exerted on proton nuclei by the N-oxyl group (see Fig. S1); moreover, once NaOH was added in the presence of HGA, peaks of the reduced form of Tempol arose (see Fig. S2), inferring the scavenger had intercepted a radical generated by HGA.

Furthermore, HGA at neutral pH was added the soluble, radical initiator 2,2′-azobis(2-methylpropionamidine) dihydrochloride (AAPH)^44^. Although with a slower rate, the same colouration develops and NMR spectra confirm the decrease in HGA concentration without the rise of other peaks.

**Figure 7:**
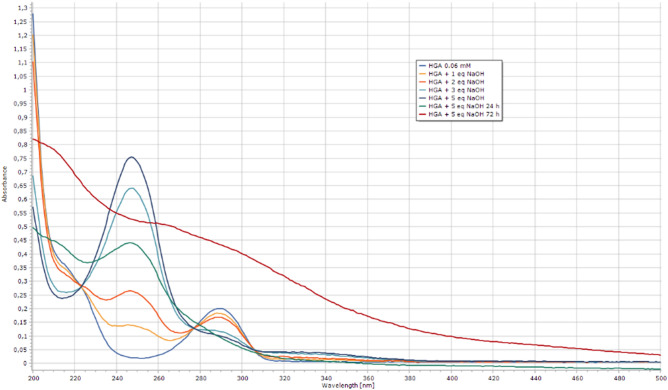
UV spectra of HGA in NaOH (0.06 mM) and the corresponding spectra after the addition of 1 to 5 equivalents of NaOH and after 24 and 72 h.

Parallel analysis of the UV spectra of HGA samples in the presence of different equivalents of NaOH was performed thus confirming the NMR data and the formation of a soluble polymer after 72 h (Fig. 7).

NMR is not suitable for the investigation of the radical species involved in the HGA transformation due to paramagnetic relaxation leading to the broadening of the signal up to complete disappearance. Electron Paramagnetic Resonance was then used to follow HGA reaction under the same condition used for NMR. The starting sample showed no paramagnetic behaviour as expected, while upon alkalinisation a strong signal appeared, as described in the following section.

### EPR monitoring of the radical transformation

**Figure 8:**
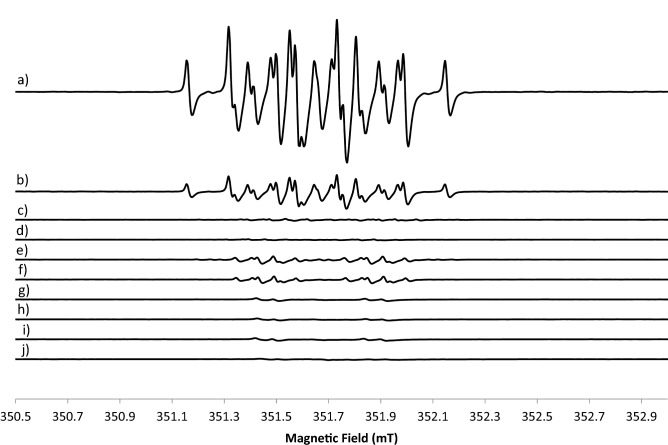
CW X-band EPR spectra at 298 K of HGA in the presence of NaOH. For the yellow solution the acquisition times are: (**a**) t = 0, (**b**) t = 5 min, (**c**) t = 20 min, (**d**) t = 4 h. For the brown solution the acquisition times are (**e**) t = 0, (**f**) t = 20 min, (**g**) t = 1 h. For the black solution the acquisition times are (**h**) t = 40 min, (**i**) t = 75 min, (**j**) t = 4 h.

To investigate reaction evolution with time, spectra were acquired at different steps. The solution becomes yellow at the beginning of the reaction and it was monitored at different times as reported in Fig. 8a–d. With time, the intensity of the signal reduces. When the colour turns to brown the lineshape of the signal changes. From few minutes to 1 h of brown colour formation, the EPR spectra were recorded and reported in Fig. 8e–g. After that, the solution becomes black. After hours of black colour the signal disappears and the corresponding spectra monitored from 40 min to 4 h are reported in Fig. 8h–j. The volumes of addition of the different reagents were set to get the highest signal possible with a longer lifetime to be able to acquire the EPR spectra at r.t.. The EPR acquisition of a black sample after a week of preparation didn’t show the presence of any paramagnetic species.

**Figure 9:**
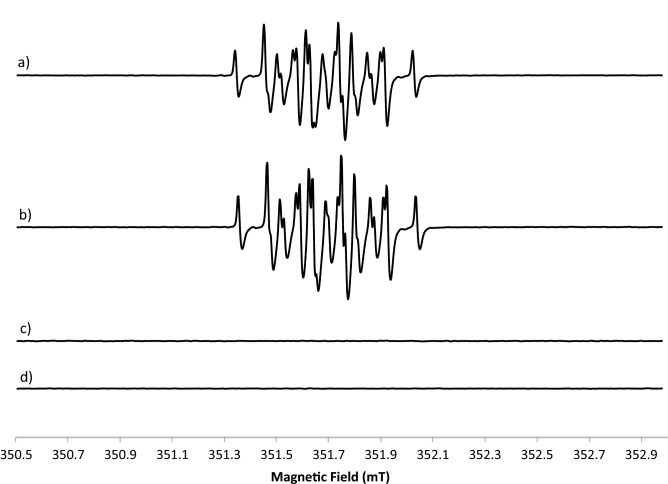
CW X-band EPR spectra at 298 K of (**a**) HGA in the presence of NaOH at t = 0, (**b**) the same of (**a**) at t = 15 min, (**c**) HGA in the presence of NaOH and treated with HCl at t = 0, (**d**) HGA in buffer at pH = 8.

To verify that the radical species formation is strongly dependent on the alkalinisation, the reaction mixture was either acidified by an HCl 1 N solution or prepared in a buffer medium at pH = 8. In Fig. 9a the EPR spectrum of HGA in the presence of NaOH at t = 0 was reported and compared with the same spectrum after 15 min (Fig. 9b). In Fig. 9c EPR spectrum obtained after the addition of HCl to the reaction mixture and in Fig. 9d EPR spectrum of the reaction performed in a buffer medium are shown. It is evident that either at acidic conditions or in the presence of a buffer the reaction doesn’t start and the radical intermediate species are not formed. Therefore, the basic conditions due to the NaOH presence is an essential requirement for the reaction to form radical species.

Due to the high complexity of the spectra of the radical species, simulation analysis was carried out to better interpret lineshape. As the spectrum of the yellow solution is very complex to be simulated because it is not perfectly symmetric and it is short-lived, simulations were started with the spectrum obtained for the black solution.

**Figure 10:**
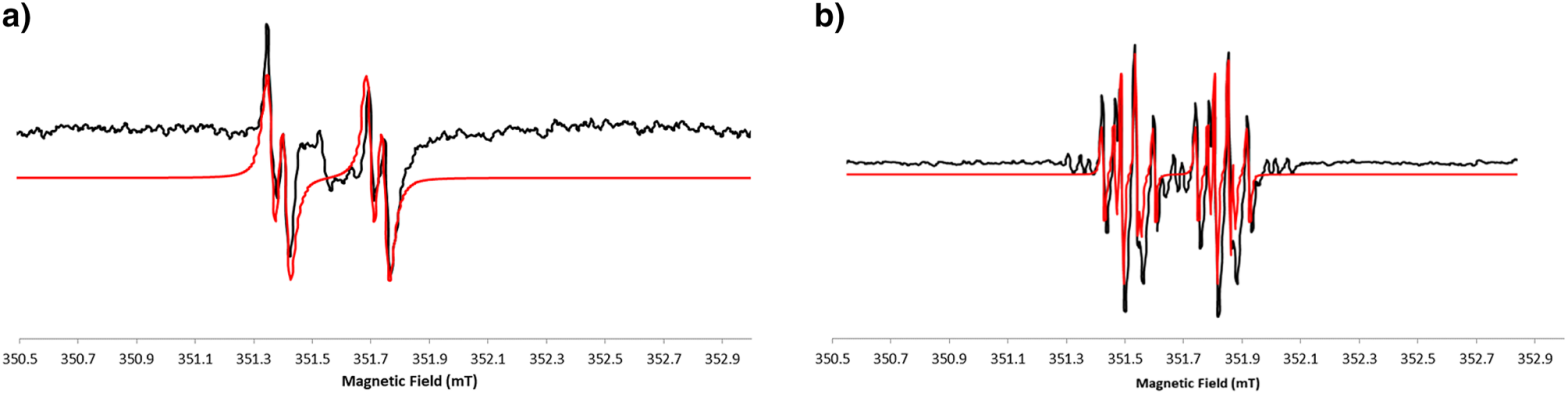
CW X-band EPR spectra at 298 K of HGA (black lines, acquired at 9.87 GHz microwave frequency, 0.1 mT modulation amplitude, 5 mW microwave power) with best-fit simulations (red lines): (**a**) in the presence of NaOH when the solution is black; (**b**) in the presence of NaOH when the solution is brown.

In Fig. 10a the EPR spectrum of the radical present in the black solution at t = 40 min is reported paired with its best fit simulation. The simulation was run with g = 2.0047 (± 0.0001) and the interaction with two H atoms was not magnetically equivalent. At first, the unpaired electron of the radical interacts with an H atom with a coupling constant A = 0.4mT and then with a second H atom with A = 0.06mT. The second spectrum simulated was that of the brown solution and it is reported in Fig. 10b. The simulation was run with a linear combination of three radical species. The first contribution derives from a radical with a g = 2.0045 (± 0.0001) and the interaction with an H atom with a coupling constant A = 0.42mT and then with a second H atom with A = 0.16mT. The second contribution derives from another radical centred at g = 2.0048 (± 0.0001) with the same number of atoms and coupling constants of the previous case (an H with A = 0.42mT and an H with an A = 0.16mT). The last species is the same present in the black spectrum characterized by a g = 2.0047 (± 0.0001) and coupling with two H atoms, one with a coupling constant A = 0.4mT and the other with an A = 0.06mT.

The simulation of the spectrum at t = 0 after the addition of the reagents when the solution is yellow was very hard due to the contemporary presence of several species that evolve over time and a complete assignment wasn’t possible. An attempt was carried out and reported in the Supporting info as Fig. S3. Since after 14 days the paramagnetic signal decreased to zero, and in the same condition also NMR shows no diamagnetic species in a significant amount, the seek for larger, slow tumbling molecules was undertaken.

### HGA transformation process ends with high molecular weight polymers

**Figure 11:**
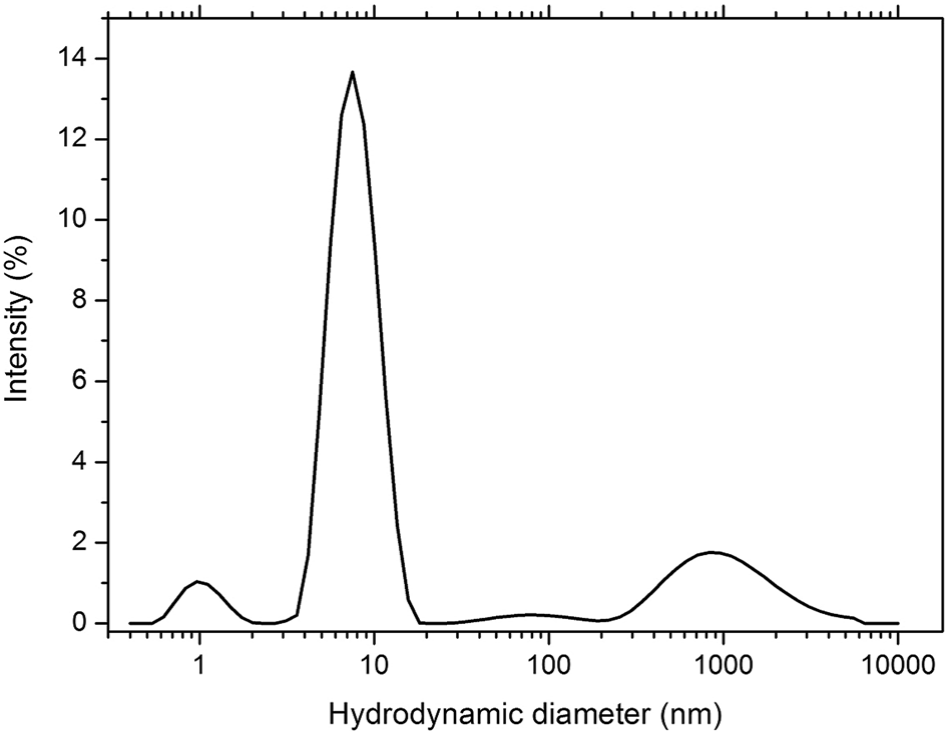
Intensity versus hydrodynamic diameter distribution curve measured for the stable, dark solution obtained by full reaction of HGA. The curve is the average of three different measurements. A prevalent contribution from species with a hydrodynamic diameter around 8 nm is apparent.

The results of the DLS measurements of the stable, dark solution obtained by full reaction of HGA are shown in Fig. 11. The intensity versus hydrodynamic diameter curve suggests that there is a rather broad size distribution of the species responsible for the scattering of the light. The population of species with a hydrodynamic diameter around 8 nm is the one contributing more to the scattered intensity. Hence, the DLS results are consistent with the formation of polymeric molecules with a broad distribution of molecular weights. A rough estimation of the average molecular weight based on the hydrodynamic diameter as determined by DLS (assuming a linear conformation of the polymer) is around 10 kDa. Such dimension, when coupled with shape factors and possibly aggregation further slowing down the molecular tumbling, may account for the missing detection by NMR spectroscopy.

### Comparison with in vitro and ex vivo models of ochronotic pigment development

The in vitro evidence of HGA fate reported in this paper for urine samples and the pure compound is consistent with previously proposed in vitro models such as human serum^45,46^ and cell cultures of osteoblastic cell lines and chondrocytes isolated from human cartilage^6,47–54^, e.g. incubation of cell cultures with HGA leads to pigment formation in 4 weeks. AKU development has also been modelled by ex vivo approaches studying the effect of HGA on human cartilage explants^49,55^, showing pigmentation to arise after 2 months of incubation with HGA. The two weeks’ time we observed is consistent with the simpler liquid phase condition used for spectroscopic studies. Whereas the development of the aforementioned models has significantly contributed to the study of other pathophysiological events associated with ochronosis such as secondary amyloidosis and oxidative stress, our approach is focused on the molecular characterization of the early radical species as well as of the large molecules that start and stop the HGA transformation process. To bridge the gap between molecular level and cell level observation further work is necessary to bring our model based on a spectroscopic approach to cell culture models.

## Conclusions

Alkaptonuria development is connected to the accumulation of HGA and subsequent deposition of ochronotic pigment in joints and cartilages. The transformation from the metabolite into the polymeric pigment has been supposed to rely on BQA as intermediate at basic pH mostly based on structural similarity between HGA and hydroquinone that is known to be oxidized to the para-benzoquinone. To test such correlation, BQA was synthesized and its behaviour under NMR acid/base titration was compared to that of HGA: alkalinisation of HGA does not lead to BQA but rather to deprotonation that turns to the loss of carbon dioxide from HGA, suggesting a free radical intermediate. Replica of the experiment with the radical scavenger Tempol substantiates such a hypothesis, and radical species formation and fate were followed by EPR. NMR also demonstrates that HGA in water solution and biofluids undergoes the same fate upon pH increase, with a decrease of the metabolite transforming into radical species as monitored by EPR and eventually into a soluble, black diamagnetic pigment, and originating a scattering compatible with a set of large molecules, whose size and associated shape possibly accounts for the missing detection in NMR. New insights on the process leading from HGA to ochronotic pigment at the molecular level have been obtained with spectroscopic evidence, also overcoming previous hypotheses relying mostly on chemical similarity. Such evidence, gained from in vitro study of HGA solution and biofluids, opens the way for molecular investigation of HGA fate in cells and tissue aiming to find new targets for Alkaptonuria therapy.

## Supplementary Information

Supplementary Information.
