# Supplementary material for: A molecular spectroscopy approach for the investigation of early phase ochronotic pigment development in Alkaptonuria
Source: Sci Rep. 2021 Nov 19;11:22562. doi: 10.1038/s41598-021-01670-z (PMC8605014; doi:10.1038/s41598-021-01670-z)
Supplement: Supplementary file 1 — Supplementary Information. [file 41598_2021_1670_MOESM1_ESM.docx]

**Supplementary Information**


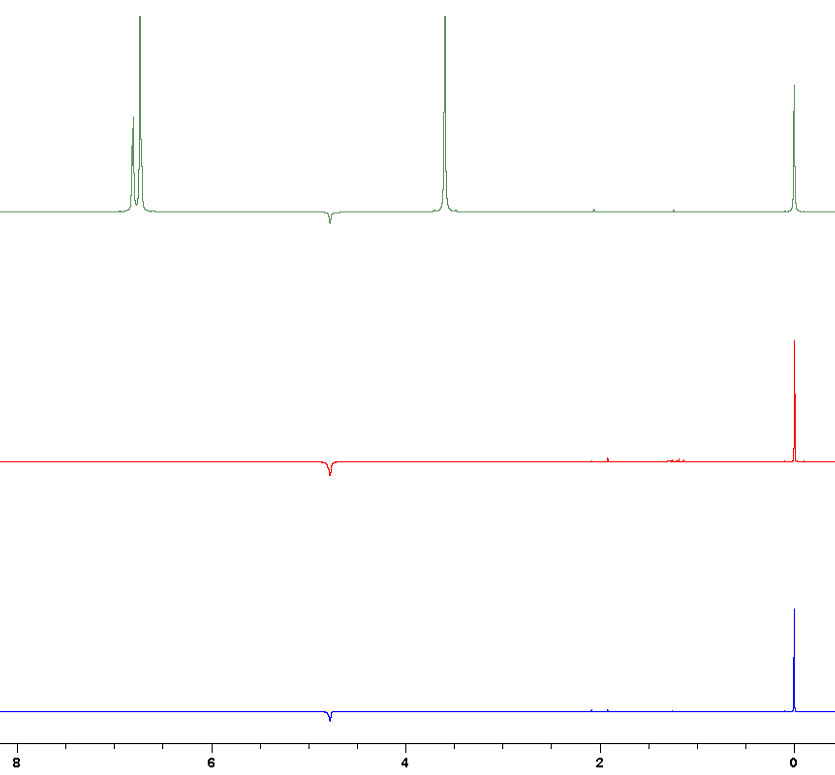


**Figure S1**. From bottom to top: Tempol acidic solution 10mM, no peak from the molecule arise due to paramagnetic relaxation; the same solution after 7 days an excess of NaOH has been added shows the molecule as stable at basic pH; the same starting solution after 7 days of HGA addition to a concentration of 5mM shows both molecules as stable (broadening of HGA signal are due to the paramagnetic solution).


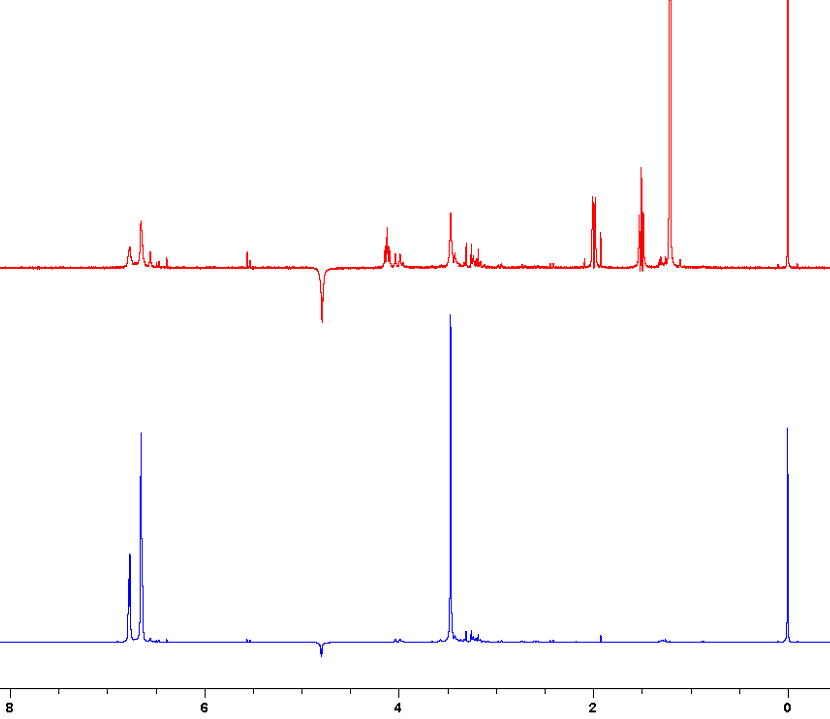


**Figure S2**. From bottom to top: HGA 5 mM solution after addition of excess of NaOH as in previous experiments, the HGA signals start decreasing: after addition of Tempol to a 10mM concentration, peaks from reduced Tempol (diamagnetic) suddenly appears, inferring the scavenger had intercepted a radical generated by HGA. The apparent signal broadening of HGA peaks is due to the excess of paramagnetic molecule.

| a) | b) |
| --- | --- |
| 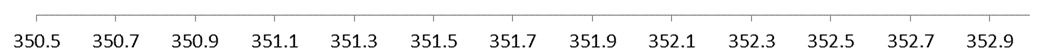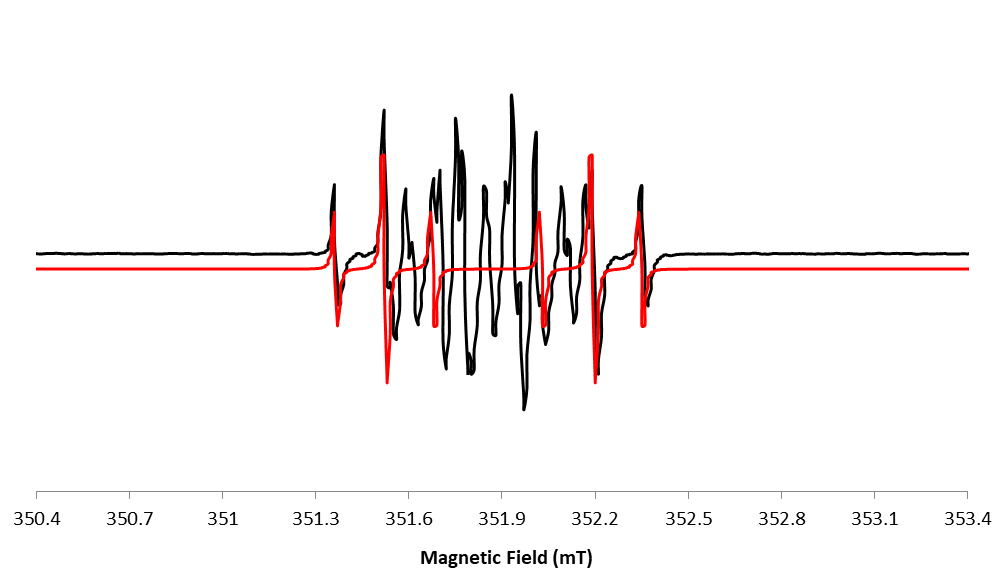 | 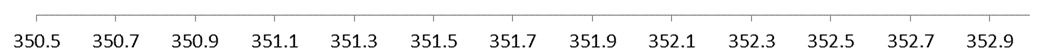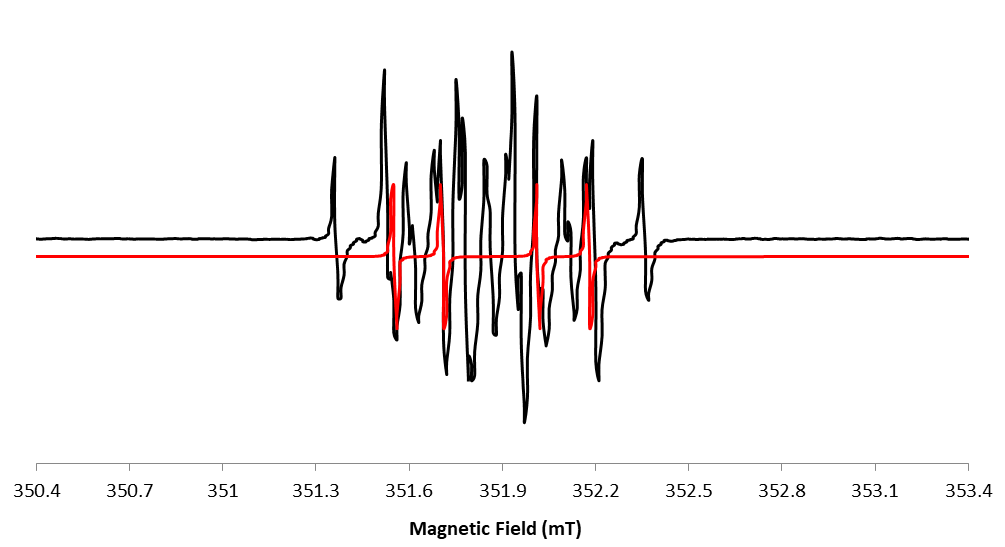 |
| c) | d) |
| 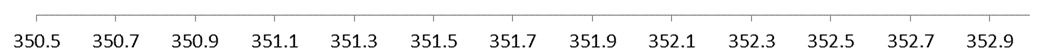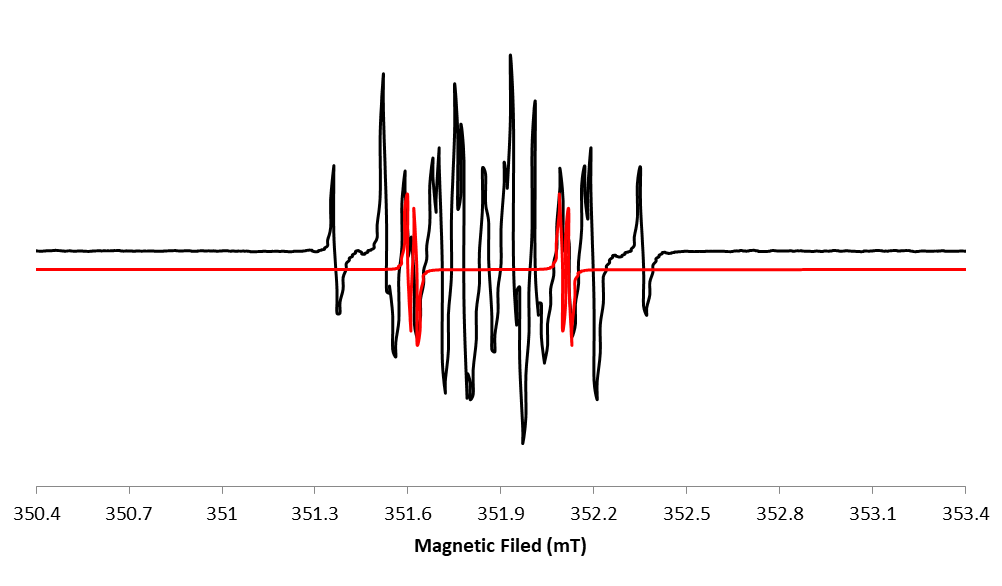 | 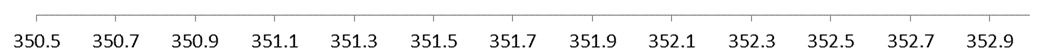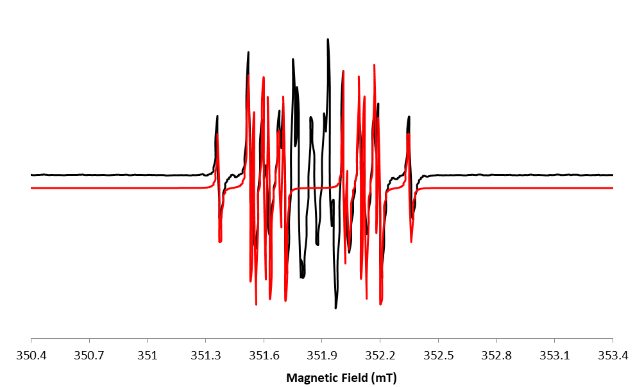 |

Figure S3. CW X-band EPR spectrum at 298K of HGA in the presence of NaOH at t=0 (yellow solution) reported with black line paired to different simulations (red line). The spectrum was acquired at 9.87 GHz microwave frequency, 0.1mT modulation amplitude, 5mW microwave power.

The first contribution (A) present at the beginning of the reaction and disappears after 20 minute sis due to a radical species characterised by a g=2.0047 (±0.0001) and by the interaction of the unpaired electron: with a H atom with a coupling constant A=0.67mT and with other two H magnetically equivalent with a coupling constant A=0.16mT. In panel a) the experimental spectrum (black line) paired to the simulation with the contribution A (red line) is shown. In panel b) the overlapping of the experimental spectrum (black line) to the simulation (red line) obtained with a g=2.0047 (±0.0001) with coupling interactions of a H with A=0.47mT and another H with A=0.16mT is reported (species B). In panel c) the experimental spectrum (black line) is paired to the simulation (red line) carried out with g=2.0049 (±0.0001) and the interaction with a H with A=0.5mT and another H with A=0.03mT (species C).

The linear combination of the last three species present in the following ratio A:B:C=1:0.6:0.6 produces the simulation (red line) paired to the experimental spectrum (black line) reported in panel d). The fitting is very good in the external sides of the spectrum but in the inside there is an asymmetric lineshape that is difficult to be simulated and is probably due to the presence of different contributions that changes during time. For that reason, even a subtraction of contributions didn't allow to assign the remaining species.

Figure S4. 1H-NMR spectrum of BQA in CD_3_OD.
